# More Than Gliding: Involvement of GldD and GldG in the Virulence of *Flavobacterium psychrophilum*

**DOI:** 10.3389/fmicb.2017.02168

**Published:** 2017-11-07

**Authors:** David Pérez-Pascual, Tatiana Rochat, Brigitte Kerouault, Esther Gómez, Fabienne Neulat-Ripoll, Celine Henry, Edwige Quillet, Jose A. Guijarro, Jean F. Bernardet, Eric Duchaud

**Affiliations:** ^1^Virologie et Immunologie Moléculaires, Institut National de la Recherche Agronomique, Université Paris-Saclay, Jouy-en-Josas, France; ^2^Área de Microbiología, Departamento de Biología Funcional, Facultad de Medicina, Instituto de Biotecnología de Asturias (IUBA), Universidad de Oviedo, Oviedo, Spain; ^3^PAPPSO, Micalis Institute, Institut National de la Recherche Agronomique, AgroParisTech, Université Paris-Saclay, Jouy-en-Josas, France; ^4^GABI, Institut National de la Recherche Agronomique, Université Paris-Saclay, Jouy-en-Josas, France

**Keywords:** *Flavobacterium psychrophilum*, fish-pathogenic bacteria, gliding motility, secretion, T9SS, virulence, *Oncorhynchus mykiss*

## Abstract

A fascinating characteristic of most members of the genus *Flavobacterium* is their ability to move over surfaces by gliding motility. *Flavobacterium psychrophilum*, an important pathogen of farmed salmonids worldwide, contains in its genome the 19 *gld* and *spr* genes shown to be required for gliding or spreading in *Flavobacterium johnsoniae*; however, their relative role in its lifestyle remains unknown. In order to address this issue, two spreading deficient mutants were produced as part of a Tn*4351* mutant library in *F. psychrophilum* strain THCO2-90. The transposons were inserted in *gldD* and *gldG* genes. While the wild-type strain is proficient in adhesion, biofilm formation and displays strong proteolytic activity, both mutants lost these characteristics. Extracellular proteome comparisons revealed important modifications for both mutants, with a significant reduction of the amounts of proteins likely transported through the outer membrane by the Type IX secretion system, indicating that GldD and GldG proteins are required for an effective activity of this system. In addition, a significant decrease in virulence was observed using rainbow trout bath and injection infection models. Our results reveal additional roles of *gldD* and *gldG* genes that are likely of importance for the *F. psychrophilum* lifestyle, including virulence.

## Introduction

Many members of the phylum *Bacteroidetes* show gliding motility, the movement of cells over surfaces without the aid of pili or flagella. This phenomenon has been studied in detail mainly in *Flavobacterium johnsoniae* (McBride and Nakane, 2015), and more recently in the marine bacterium *Cellulophaga algicola* (Zhu and McBride, 2016). The components involved in the gliding process have been identified by screening for gliding defects using transposition mutant libraries in *F. johnsoniae*. Twelve *gld* genes (*gldA, gldB, gldD, gldF, gldG, gldH, gldI, gldJ, gldK, gldL, gldM, gldN*) are required for gliding, seven *spr* genes (*sprA, sprB, sprC, sprD, sprE, sprF*, and *sprT*) are involved in colony spreading but dispensable for cell individual movement, and several *rem* genes encode proteins with redundant motility functions (Hunnicutt et al., 2002; Braun and McBride, 2005; Braun et al., 2005; Liu et al., 2007; Nelson et al., 2007, 2008; Rhodes et al., 2011a,b; Shrivastava et al., 2012). Strikingly, some of these genes (i.e., *gldK, gldL, gldM, gldN, sprA, sprE*, and *sprT*) are orthologs of *porK, porL, porM, porN, sov, porW*, and *porT* genes, respectively, encoding the core secretion machinery of the newly described Type IX secretion system (T9SS) identified in the non-gliding periodontal pathogen *Porphyromonas gingivalis* (Sato et al., 2010, 2013). Additional components of T9SS have been also identified such as PorP, the PorU signal peptidase (Glew et al., 2012), PorV (Kharade and McBride, 2015), the PG1058 lipoprotein (Heath et al., 2016), and the PorZ surface component (Lasica et al., 2016), for which the exact roles in protein secretion remain unknown. Most of the T9SS proteins showed homologs only in *Bacteroidetes* genomes such as those of *Flavobacterium, Capnocytophaga, Cellulophaga, Cytophaga*, and *Tannerella* species, suggesting that this transport system is apparently restricted to this phylum (McBride and Zhu, 2013). It has been demonstrated that the T9SS is required for the secretion, cell surface exposition, attachment, or the external release of proteins with various functions in diverse *Bacteroidetes* species (Sato et al., 2010; Shrivastava et al., 2013; Narita et al., 2014; Tomek et al., 2014; Zhu and McBride, 2014; Kita et al., 2016). Moreover, most of these proteins secreted by the T9SS possess conserved C-terminal domains (CTDs) required for their translocation across the outer membrane. These 70–100 amino acids long CTDs mainly belong to the TIGR04183 or TIGR04131 protein domain families (McBride and Nakane, 2015; Kulkarni et al., 2017). However, other T9SS-mediated proteins have been identified, such as the *F. johnsoniae* chitinase ChiA, that display different CTDs in their sequence (Kharade and McBride, 2014). Importantly, motility and secretion systems appear to be intertwined since it has been shown that the T9SS is essential for the secretion of several surface-exposed motility adhesins in *F. johnsoniae* (Rhodes et al., 2011b; Shrivastava et al., 2013) and *Capnocytophaga ochracea* (Kita et al., 2016). Indeed, some *F. johnsoniae* adhesins are important for gliding. They are rapidly propelled along the cell surface by the rest of the motility machinery (Nakane et al., 2013; Shrivastava et al., 2015). This process appears to be driven by a proton-motive force-dependent trans-envelope motor (Nakane et al., 2013; McBride and Nakane, 2015; Shrivastava and Berg, 2015; Shrivastava et al., 2015).

*Flavobacterium psychrophilum* is an important fish pathogen. This bacterium is the etiologic agent of rainbow trout fry syndrome (RTFS) and bacterial cold-water disease (BCWD), two conditions of utmost significance for freshwater-reared salmonids. Outbreaks occur at temperatures below 14°C and cause important economic losses for salmonid fish farms worldwide (Nematollahi et al., 2003a; Starliper, 2011). Despite extensive research, no commercial vaccine against the infections provoked by *F. psychrophilum* is available, except in Chile, resulting in the administration of antibiotics to treat outbreaks (Gómez et al., 2014). Furthermore, the mechanisms of pathogenicity of this microorganism are still poorly understood (Álvarez et al., 2006, 2008; Pérez-Pascual et al., 2011, 2015; Nakayama et al., 2015). Several improvements have been reported during the last decades in bacterial physiology (Álvarez et al., 2004; Pérez-Pascual et al., 2009), molecular diagnosis (Cepeda and Santos, 2000; del Cerro et al., 2002; Fujiwara-Nagata and Eguchi, 2009; Strepparava et al., 2014), molecular epidemiology (Nicolas et al., 2008; Siekoula-Nguedia et al., 2012; Fujiwara-Nagata et al., 2013; Avendaño-Herrera et al., 2014; Nilsen et al., 2014; Van Vliet et al., 2016; Ngo et al., 2017), genome analysis (Duchaud et al., 2007; Wiens et al., 2014; Wu et al., 2015; Rochat et al., 2017a,b), and development of genetic tools (Álvarez et al., 2006; Pérez-Pascual et al., 2011; Gómez et al., 2012, 2015), opening the way for functional genomics studies.

Gliding motility has not been previously studied in detail in *F. psychrophilum*. Analyses of *F*. *psychrophilum* genomes revealed that all the above-mentioned gliding genes as well as T9SS-encoding genes studied in *F. johnsoniae* or *P. gingivalis* so far are well-conserved (Duchaud et al., 2007; Rochat et al., 2017a). With the aim of achieving a deeper insight into these two intertwined biological processes, as well as their relevance into the pathogenesis of *F. psychrophilum*, a set of mutants deficient in spreading were isolated using Tn*4351*-mutagenesis in strain THCO2-90. Using *in vitro* and *in vivo* phenotyping as well as proteomics, we performed an exhaustive analysis of two of these mutants and identified important defects in extracellular proteolytic activities, adhesion, biofilm formation, and exoproteome composition. Importantly, these mutations provoked a high attenuation of the virulence of *F. psychrophilum* in rainbow trout (*Oncorhynchus mykiss*), a natural-host infection model.

## Materials and methods

### Bacterial strains and growth conditions

The strains, plasmid and primers used in this study are listed in Table 1. *Escherichia coli* strains S17-1, BW19851 (Metcalf et al., 1994) or MFD*pir* (Rochat et al., 2017b) were used to transfer DNA into *F. psychrophilum* THCO2-90 by conjugation. *E. coli* strains were grown at 37°C in Luria Bertani (LB) with 15 g of agar per liter added for solid medium. *F. psychrophilum* THCO2-90 was grown at 18°C in tryptone yeast extract salts (TYES) broth [0.4% (w/v) tryptone, 0.04% yeast extract, 0.05% (w/v) MgSO_4_ 7H_2_O, 0.02% (w/v) CaCl_2_ 2H_2_O, 0.05% (w/v) D-glucose, pH 7.2] or in modified Bushnell-Haas broth (BH; Sigma-Aldrich Co.) supplemented with a vitamin cocktail at pH 7 (5 mg L^−1^ pyridoxamine, 1 mg L^−1^ nicotinic acid, 1 mg L^−1^ thiamine, 1 mg L^−1^ riboflavine, 1 mg L^−1^ D,L-panthotenic acid, 10 mg L^−1^ 4-aminobenzoic acid, 1 mg L^−1^ D-biotine, 1 mg L^−1^ folic acid, 1 mg L^−1^ vitamin B12, 5 mg L^−1^ orotic acid anhydrous, 5 mg L^−1^ thymidine, 5 mg L^−1^ inosine and 2.5 mg L^−1^ thioctic acid), 100 μM FeCl_3_, 50 μM CaCl_2_ and 0.5% (w/v) casein or gelatin when needed. Growth in liquid culture was carried out at 200 rpm and 18°C and evaluated by measuring OD_600 nm_ at different times. Stock cultures were preserved in TYES broth containing 20% (v/v) glycerol at −80°C. To observe colony spreading, *F. psychrophilum* strains were grown on 1/5 TYES with 15 g L^−1^ of agar (Pérez-Pascual et al., 2009). Extracellular proteolytic activity on solid medium was visualized by using TYES containing 15 g L^−1^ of agar and supplemented with 0.75% (w/v) gelatin or casein (Álvarez et al., 2006). For selective growth of *E. coli* strains carrying pEP4351 and pCP-derivative plasmids, transformants were selected with 20 μg mL^−1^ chloramphenicol and 100 μg mL^−1^ ampicillin, respectively. Cultures of *E. coli* MFD*pir* were supplemented with 0.3 mM diaminopimelic acid (Sigma-Aldrich Co.). Selection of *F. psychrophilum* transconjugants was carried out with 10 μg mL^−1^ of gentamycin or erythromycin.

**Table 1:** Bacterial strains, plasmids, and primers used in this study.

**Plasmid, strain, primer**	**Description/Sequence (5′ -> 3′)**	**Source or References**
**PLASMIDS**		
pEP4351	*Ori* R6K dependent protein pir,; RP4 oriT; Cm^r^ Tc^r^ (Em^r^); Tn*4351* vector transfer	Cooper et al., 1997
pCP23	*E. coli*–*F. psychrophilum* shuttle plasmid; ColE1 ori (pCP1 ori), Ap^r^ (Tc^r^)	Agarwal et al., 1997
pCP*Gm^*R*^*	pCP23-derivative carrying *aac(6′)-aph(2′)* gentamycin resistance gene; Ap^r^ (Gm^r^)	This study
pCP*Gm^*R*^-gldD*	pCP*Gm^*R*^*-derivative carrying P*_*orf*1_*-*gldD;* Ap^r^ (Gm^r^)	This study
pCP*Gm^*R*^-gldG*	pCP*Gm^*R*^*-derivative carrying P*_*orf*1_*-*gldG;* Ap^r^ (Gm^r^)	This study
***F. psychrophilum*** **STRAINS**		
OSU THCO2-90	*F. psychrophilum* isolated from Coho salmon	Bertolini et al., 1994
TRV107	THCO2/90 *gldD::*Tn*4351*; (Em^r^)	This study
TRV103	THCO2/90 *gldG::*Tn*4351;* (Em^r^)	This study
TRV272	THCO2/90 pCP*Gm^*R*^* (Gm^r^)	This study
TRV323	THCO2/90 *gldD::*Tn*4351* pCP*Gm^*R*^* (Em^r^; Gm^r^)	This study
TRV339	THCO2/90 *gldG::*Tn*4351* pCP*Gm^*R*^* (Em^r^; Gm^r^)	This study
TRV329	THCO2/90 *gldD::*Tn*4351* pCP*Gm^*R*^*-*gldD* (Em^r^; Gm^r^)	This study
TRV338	THCO2/90 *gldG::*Tn*4351* pCP*Gm^*R*^*-*gldG* (Em^r^; Gm^r^)	This study
***E. coli*** **STRAINS**		
S17-1	*recA pro hsdR* RP4-2(Tc^r^::Mu-Km^r^::Tn7 Str^r^)	Simon et al., 1983
MFD*pir*	MG1655 RP4-2-Tc::[ΔMu1::*aac(3)IV*-Δ*aphA*-Δ*nic*35-ΔMu2::*zeo*] Δ*dapA*::(*erm*-*pir*) Δ*recA*	Rochat et al., 2017b
BW19851	RP4-2(*tet*::Mu-1*kan*::Tn*7* integrant) *uidA*::*pir recA1 hsdR17 creB510 endA1 zbf-5 thi*	(Metcalf et al., 1994)
**PRIMERS**		
TN-1	GGACCTACCTCATAGACAA	
IS4351-F	TCAGAGTGAGAGAAAGGG	
TRO300	TTGGATTAAGCAATAATATACTACAATAGATGC	
TRO301	TAATGGAGCGGTCAGGAAAT	
TRO302	GCATCTATTGTAGTATATTATTGCTTAATCCAAATGAATATAGTTGAAAATGAAAT	
TRO303	ATTTCCTGACCGCTCCATTAATCTTTATAAGTCCTTTTATAAATT	
TRO308	GTTCTCATATGCTACGAGGAGG	
TRO319	GTACTGAGAGTGCACCATACGTC	
TRO370	TTTGAGGGATAATAAAAAGGATAATTATGTTTAATAAATATATTACTTCTCTTTT	
TRO371	CGGTCCGGAATTCCCTATAACAGATAACGGACAAAAACTTC	
TRO372	TTTGAGGGATAATAAAAAGGATAATTATGATACCAATTAAGAAAAAGAAAATC	
TRO373	CGGTCCGGAATTCCCTATAATCGTAAACTAATCAGTATTGAAAACAT	
TRO350	AATTATCCTTTTTATTATCCCTCAAA	
TRO351	TTATAGGGAATTCCGGACCG	
TRO137	GAGGGAACGACGCAAAGCGATAGTTC	
TRO138	GGAAACAGCTATGACCATGATTACGCC	

*Underlined sequences correspond to regions which hybridize with targeted genes [i.e., aac(6′)-aph(2′) gentamycin resistance marker, gldD, and gldG genes]*.

### DNA technology

Genomic DNA extraction was performed with the Gen Elute Bacterial DNA (Sigma-Aldrich Co.) extraction kit. Plasmid DNA was purified with the NucleoSpin® Plasmid (Machery-Nagel) kit. PCR amplification products were separated on 1% agarose gels and bands were purified with the Illustra^TM^ GFX, PCR DNA and the Gel Band Purification Kit gel extraction system.

### Tn*4351* mutant library construction

*E. coli* BW19851 was used for conjugative transfer of pEP4351 plasmid carrying Tn*4351* into *F. psychrophilum* strain THCO2-90 as previously described (Álvarez et al., 2004). Briefly, the donor *E. coli* strain was grown to mid-log phase in LB broth and 10 mL were centrifuged to harvest cells. Cells were washed twice with TYES broth, and suspended in 50 μL of TYES broth. The recipient *F. psychrophilum* strain was grown to mid-log phase in TYES broth, 10 mL of culture was centrifuged, and the cell pellet was washed twice with TM buffer, consisting of 20 mM Tris-HCl and 20 mM MgSO_4_ pH 7.2, and suspended in 50 μL of TM buffer. Cell suspensions of *F. psychrophilum* and *E. coli* were mixed together, spotted onto TYES agar, and incubated at 20°C for 48 h. After conjugation, cells were scraped off the plates, diluted in 1 mL of TYES broth, and plated on TYES agar containing 10 μg mL^−1^ erythromycin. Erythromycin-resistant colonies of *F. psychrophilum* appeared on TYES agar after 5–7 days of incubation at 18°C.

### Identification of Tn*4351* interrupted locus and sequencing of the surrounding DNA region

The insertion of Tn*4351* into the genome of 439 transconjugants was firmly established using inverse-PCR as previously described (Álvarez et al., 2006). Briefly, genomic DNA of the mutant strains was digested with *Hind*III followed by a re-ligation process. The resulting circular molecules were used as a template to amplify by inverse PCR the sequences adjacent to the Tn*4351* insertion site using a specific pair of primers TN-1/IS4351-F (Table 1) and the GoTaq DNA polymerase (Promega, France). Sanger sequencing of the PCR amplified products was performed on an ABI PRISM 3100 (Applied Biosystems, CA, USA) and sequences were used to locate the transposon insertion site on the THCO2-90 genome (Rochat et al., 2017a).

### Construction of pCP*Gm^*R*^* shuttle vector, a pCP23-derivative vector carrying a gentamycin resistance marker

The coding sequence of *tetQ* was replaced by *aac(6*′*)-aph(2*′*)* gene encoding a gentamycin resistance marker, while keeping expression signals unchanged (plasmid map in Figure S1). pCP*Gm*^*R*^ was constructed as follows: the two DNA fragments were amplified by PCR using Phusion High-Fidelity DNA polymerase (Thermo Fisher), the vector fragment using pCP23 DNA as matrix and primers TRO300/TRO301, *aac(6*′*)-aph(2*′*)* using pZXL5 DNA (Zhang et al., 2012) and primers TRO302/TRO303. The resulting PCR products were assembled by the method developed by Gibson (Gibson et al., 2009) using the Gibson Assembly Master Mix (New England Biolabs). Engineered plasmids were constructed in *E. coli* S17-1. Correct replacement of *tetQ* by *aac(6*′*)-aph(2*′*)* was verified by PCR and DNA sequencing with primers TRO308 and TRO319. pCP*Gm*^*R*^ was then transferred by electrotransformation to *E. coli* MFD*pir*, the donor strain used subsequently to introduce plasmids into *F. psychrophilum* by conjugation. Transconjugants were selected on TYES agar supplemented with 10 μg mL^−1^ gentamycin and appeared between 3 and 5 days of incubation at 18°C. The presence of plasmid was checked by plasmid DNA extraction.

### Complementation of *gldD* and *gldG* mutants

Two pCP*Gm*^*R*^ derivative plasmids containing the coding sequence of *gldD* and *gldG* were constructed using Gibson's method (plasmid map available in Figure S2). Briefly, DNA sequences of interest were amplified from THCO2-90 genomic DNA by PCR using primers TRO370/TRO371 and TRO372/TRO373 for *gldD* and *gldG*, respectively. The vector was amplified by PCR using primers TRO350/TRO351 and pCP*Gm*^*R*^ as DNA matrix. The DNA assembly results in the insertion of *gld* gene upstream of the expression signals of ORF1 of pCP1, a cryptic plasmid isolated from a *F. psychrophilum* isolate (McBride and Kempf, 1996). Resulting plasmids pCP*Gm*^*R*^–*gldD* and pCP*Gm*^*R*^–*gldG* were checked by PCR and DNA sequencing with primers TRO137 and TRO138. Plasmids were transferred into relevant *F. psychrophilum* strains by conjugation with *E. coli* MFD*pir*.

### Adhesion and biofilm assays

Adhesion ability of each strain to 96-well microtiter polystyrene plates with flat bottom (Nunclon^TM^ Delta surface, Nunc) was evaluated as previously described (Högfors-Rönnholm et al., 2015) with few modifications. Briefly, the wild-type strain THCO2-90 and the two mutant strains were grown in TYES broth to OD_600nm_ = 0.5. One milliliter of each bacterial culture was centrifuged at 11,093 g for 5 min, the supernatant was removed and cells pellet was resuspended in sterile distilled water. One hundred of microliters of each bacterial suspensions were added in quadruplicate to the microplate and incubated at 18°C for 3 h without shaking. Then, wells were washed twice with sterile distilled water. The adherent cells were stained with 100 μL 1% (w/v) crystal violet solution for 30 min at room temperature. Excess stain was removed by washing the wells four times with sterile distilled water, and stain bound to the adherent cells was released with 100 μL absolute ethanol for crystal violet solubilization. The adhesion ability of the bacterial cells was determined by measuring the OD_595nm_ using a Tecan Microplate Reader (Infinite 200 PRO). As a negative control non-inoculated sterile milliQ water was used. Percentages of binding refer to the level of adhesion observed for each strain, compared with adhesion of the wild-type strain which higher OD_595nm_ value was set to 100%. All assays were performed in quadruplicate and repeated at least two times for reproducibility.

Biofilm formation was evaluated using the standard assay with crystal violet staining as previously described for *F. psychrophilum* (Álvarez et al., 2006; Levipan and Avendaño-Herrera, 2017) with some modifications. The wild-type strain THCO2-90 and the two mutant strains were grown in half-strength TYES broth to the mid-exponential phase. The cultures were diluted 1:100 in half-strength TYES broth, and 150 μL were deposited in wells of 96-well microtiter polystyrene plates with flat bottom (Nunclon^TM^ Delta surface, Nunc). Wells containing non-inoculated medium were used as negative controls. The plate was incubated at 18°C under static condition for 120 h. Every 24 h, biofilm development was evaluated in four wells by strain. The supernatants were discarded, the wells were washed twice with 200 μL of sterile distilled water then 150 μL of 1% (w/v) crystal violet was added to each well. After 30 min, excess stain was removed by washing the wells four times with 200 μL of sterile distilled water and the stain bound to adherent cells was subsequently released by adding 100 μL of absolute ethanol. The biofilm formation was determined by measuring the OD_595 nm_ using a Tecan Microplate Reader and quantified as the specific biofilm formation (SBF) index proposed by (Niu and Gilbert, 2004): SBF = (B − NC)/G, where *B* is the amount of ethanol-solubilized crystal violet released from biofilm cells, *NC* is the amount of ethanol-solubilized crystal violet adherent to wells of negative controls, and *G* is the absorbance of the cell supernatant.

### Extracellular protein analysis by LC-MS/MS

Cultures of *F. psychrophilum* strains THCO2-90, *gldD::Tn* and *gldG::Tn* were grown in 50 mL of TYES broth at 200 rpm and 18°C until reaching late exponential phase (OD_600_ = 0.8). For secretome analysis, the supernatants were recovered by centrifugation at 6,000 g for 10 min at 4°C and concentrated by ultrafiltration using Amicon Ultra-centrifugal filters (Millipore, MW cut off 10 kDa) at 4,000 g for 30 min at 4°C. Then, 10 μg of each protein suspension was separated using one-dimensional short migration in SDS-PAGE. In-gel digestion of the proteins was performed on bands excised from one-dimensional SDS-PAGE. Each lane of short migration was cut and washed for 15 min with an acetonitrile/100 mM ammonium bicarbonate mixture (1:1). Digestion was performed in 50 mM ammonium bicarbonate pH 8.0 and the quantity of modified trypsin (Promega, sequencing grade) was 0.1 μg per sample. Digestion was carried out for 6 h at 37°C. The supernatant was reserved. Peptides were extracted by 5% formic acid in water/acetonitrile (v/v). Supernatant and extracted tryptic peptides were dried and resuspended in 50 μL of 0.1% (v/v) formic acid and 2% (v/v) acetonitrile. For shaving, cells pellets obtained from the culture centrifugation were washed twice in PBS and resuspended in 0.8 mL of PBS containing 1.2 mM sucrose and 1 mM CaCl_2_ pH 7.4. Samples were digested in-solution for 10 min at 37°C by adding 2 μg mL^−1^ of sequencing-grade modified trypsin. Enzymatic reaction was quenched by reducing the pH of peptide mixtures with 0.1% formic acid. Samples were filtered with Millex GV (0.25 μm, Millipore, ref SLCG004SL) and the resulting peptide mixtures were pre-cleaned with a Strata-X column (Phenomenex, ref. 8B-S100-TAK). Columns were washed with 1.5 mL of washing buffer [3% acetonitrile (ACN) and 0.1% trifluoroacetic acid (TFA)]. The peptide mixtures were charged into the columns, followed by three washing steps of 500 μL. Elution of peptides was achieved using 600 μL of elution buffer (40% ACN and 0.1% TFA). The resulting samples were concentrated under vacuum to dryness and resuspended in 50 μL of 0.1% TFA and 2% ACN. Before analysis in a high-resolution mass spectrometer, samples were diluted 1/100.

LC-MS/MS analysis was performed using an Ultimate 3000 RSLC system (Dionex, Voisins-le-Bretonneux, France) connected to a LTQ Orbitrap mass spectrometer (Thermo Fisher) by a nanoelectrospray ion source. Samples were resuspended in 50 μL of nano HPLC buffer (2% ACN/ 0.1% formic acid) and a dilution 1/100 was achieved for shaving experiments.

### Liquid chromatography—mass spectrometry

Mass spectrometry was performed using an Orbitrap Fusion™ Lumos™ Tribrid™ (Thermo Fisher Scientific) coupled to an UltiMate™ 3000 RSLCnano System (Thermo Fisher Scientific). Four microliters of each sample were loaded at 20 μL min^−1^ on a precolumn (μ-Precolumn, 300 μm i.d × 5 mm, C18 PepMap100, 5 μm, 100 Å, Thermo Fisher) and washed with loading buffer. After 3 min, the precolumn cartridge was connected to the separating column (Acclaim PepMap®, 75 μm × 500 mm, C18, 3 μm, 100 Å, Thermo Fisher). Buffer A consisted of 0.1% formic acid in 2% acetonitrile and buffer B of 0.1% formic acid in 80% acetonitrile.

The peptide separation analysis was achieved at 300 nl min^−1^ with a linear gradient from 1 to 35% buffer B for 50 min and 35 to 45% for 5 min. One run took 65 min, including the regeneration step at 98% buffer B. Ionization (1.6 kV ionization potential) and capillary transfer (275°C) were performed with a liquid junction and a capillary probe (SilicaTip™ Emitter, 10 μm, New Objective). Peptide ions were analyzed using Xcalibur 3.1.66.10. The machine settings were as follows: 1) full MS scan in Orbitrap (scan range [m/z] = 400–1,500) and 2) MS/MS using CID (35% collision energy) in Orbitrap (AGC target = 4.0 × 10^2^, max. injection time = 50 ms, data type = profile). Analyzed charge states were set to 2–5, the dynamic exclusion to 60 s and the intensity threshold was fixed at 5.10^3^.

### Processing and bioinformatics analyses

The genome of *F. psychrophilum* THCO2-90 (Rochat et al., 2017a) was searched by the X!TandemPipeline (open source software developed by PAPPSO, version 3.4.3, http://pappso.inra.fr/bioinfo/xtandempipeline/). Protein identification was run with a precursor mass tolerance of 10 ppm and a fragment mass tolerance of 0.5 Da. Enzymatic cleavage rules were set to trypsin digestion (“after Arg and Lys, unless Pro follows directly after”) and no semi-enzymatic cleavage rules were allowed. The fix modification was set to cysteine carboxyamidomethylation and methionine oxidation was considered as a potential modification. In a second pass, N-terminal acetylation was added as another potential modification, whereas all other previous settings were retained. The identified proteins were filtered as follows: 1) peptide *E* < 0.01 with a minimum of 2 peptides per protein and 2) a protein *E* < 10^−4^.

### Statistical analysis of LC-MS/MS data

Peptide quantities of the proteome were analyzed by spectral counting (SC). SC takes into account the number of assigned spectra for each protein and is correlated to relative protein abundance. The *P*-values obtained from both ANOVA for the SC were considered significant below a value of 0.01.

### Fish infection challenges

The rainbow trout (*O. mykiss*) homozygous line A36 was used (Quillet et al., 2007). The uniformity of genetic background in isogenic lines and the high susceptibility of line A36 to *F. psychrophilum* infection makes this line highly relevant to test for changes in bacterial virulence. Fish were reared at 10°C in dechlorinated recirculated water until they reached 3–4 g, and were then transferred to continuous flow aquaria for infection experiments. Bacteria used for infections were prepared as follows: strains THCO2-90, *gldD::Tn* and *gldG::Tn* were grown in TYES broth at 200 rpm and 18°C until late-exponential phase (DO_600 nm_ = 1). This culture density corresponds to 10^9^ colony-forming units (CFU) mL^−1^, determined by serial dilutions and plate counting on TYES agar. Two experimental infection models differing by the infection route were tested: intramuscular injection and immersion. Two independent experiments were performed for each experimental infection model.

For injection challenge, 50 μL of serial dilutions performed in TYES broth by diluting bacterial cultures to obtain 10^6^, 10^7^, and 10^8^ CFU mL^−1^ were used. These doses correspond to theoretical 0.1, 1, and 10 LD_50_ previously determined for the wild-type strain. Groups of 10 fish were challenged with each dose by intramuscular injection after anesthesia. As a negative control, a group of 10 fish were injected with 50 μL of sterile TYES broth.

For immersion challenge, bacterial cultures performed in TYES broth were diluted directly into the water of aquaria (15 L) at a final concentration of 5 × 10^6^ CFU mL^−1^. Bacteria were maintained in contact with fish for 24 h by stopping the water flow then subsequently removed by restoring the water flow. Sterile TYES broth was used for the control group. Bacterial counts were determined at the beginning and at the end of the immersion challenge by plating serial dilutions of water samples on TYES agar. Water was maintained at 10°C and under continuous oxygenation for the duration of the immersion. Groups were composed of 46 fish. Virulence was evaluated according to fish mortality 14 days post-infection. Six fish of each group were randomly chosen and sacrificed 6 h after the end of immersion challenge to evaluate the bacterial load from spleen, gill, and skin mucus. Organs were mechanically disrupted in Lysing Matrix tubes containing 500 μL of 1% peptone water and 1 mm ceramic beads (Mineralex). Samples were homogenized at 6.0 m s^−1^ for 45 s on a FastPrep-24 instrument (Thermo Fisher). Serial dilutions of the homogenized solution were plated on TYES agar.

### Ethics statements

Animal experiments were performed in accordance with the European Directive 2010/2063/UE and approved by the institutional review ethics committee, COMETHEA, of the INRA Center in Jouy-en-Josas, France. Authorizations were approved by the Direction of the Veterinary Services of Versailles (authorization number 15-58).

## Results

### Isolation of Tn*4351 gldD* and *gldG* mutants

**Figure 1:**
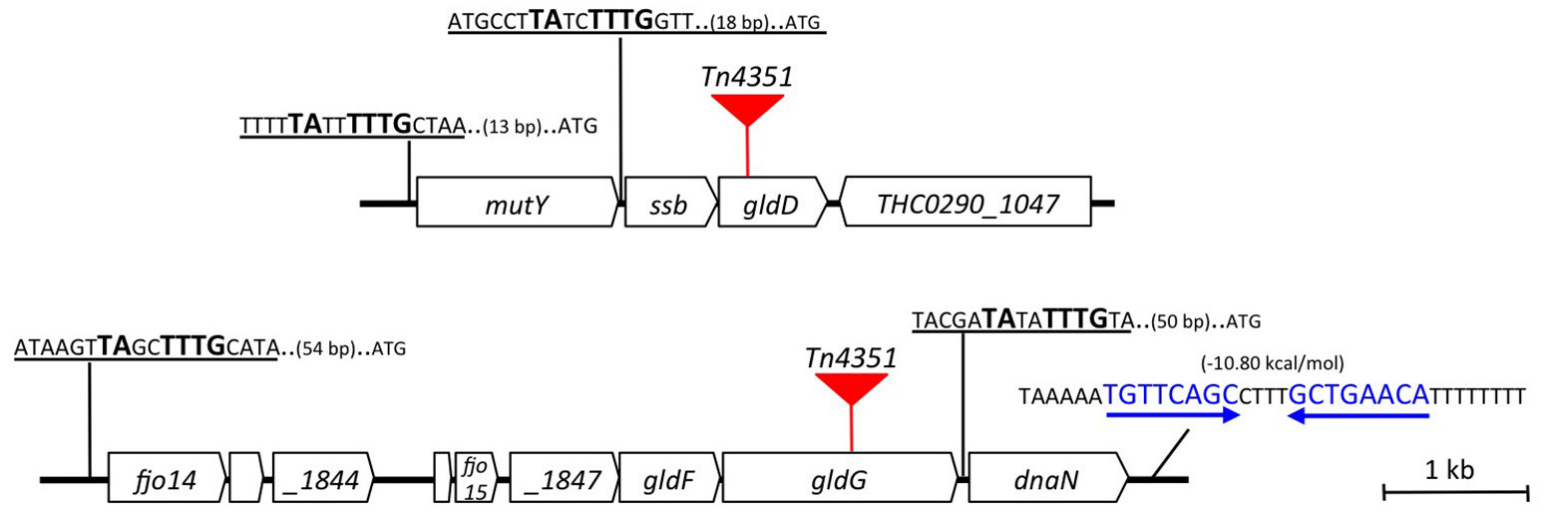
Map of the genetic context of *gldD::Tn* and *gldG::Tn* mutants. Red triangles correspond to Tn*4351* insertion in the *gldD* (upper panel) and *gldG* (lower panel) genomic regions. Putative promoter sequences are underlined, the consensus sequence of *Bacteroidetes* promoters (“TAnnTTTG” box) are shown in bold. Putative Rho-independent terminators, shown in blue, were predicted using ARNold finding terminators (http://rna.igmors.u-psud.fr/toolbox/arnold/).

To develop a functional genomic approach aiming to understand the role and the relative importance of genes of *F. psychrophilum*, a Tn*4351-*mutant library was constructed in strain THCO2-90 according to the previously developed strategy (Álvarez et al., 2004). About 2,000 erythromycin-resistant transconjugants were obtained. The specific localization of Tn*4351* was performed by inverse PCR (Álvarez et al., 2006) on a subset of this library, formally identifying the insertion site for 439 transconjugants. Among them, two strains carrying a transposon insertion into *gld* homologous genes were selected for further characterization (Figure 1). In the first one, hereafter named *gldD::Tn*, the Tn*4351* transposon is located after position 1,222,107 of the THCO2-90 chromosome and interrupts the *THC0290_1046* gene, disrupting the protein after amino acid residue 36 (out of a total of 187). *THC0290_1046* encodes the gliding motility lipoprotein precursor GldD (protein_id = SHH90844.1). In the other mutant, hereafter named *gldG::Tn*, the Tn*4351* transposon is located after position 2,148,164 and disrupts the *THC0290_1849* gene that encodes the gliding motility transmembrane protein GldG (protein_id = “SHI04365.1”), leading to a truncated protein after residue 307 (out of a total of 559). GldG likely forms along with GldA and GldF an ATP-dependent ABC transporter, found to be required for gliding in *F. johnsoniae* (Hunnicutt and McBride, 2001). *In silico* analysis of their genetic context suggests that *gldD* could be transcribed from the promoters of *mutY* and *ssb* genes (Figure 1). *gldG* lies downstream of *gldF*, a genetic organization highly conserved among genomes of the class *Flavobacteriia* carrying *gldG* and *gldF* homologous genes (Figure S3; McBride and Zhu, 2013) and upstream of *dnaN*, an essential gene likely transcribed from its own promoter (Figure 1).

### GldD and gldG are involved in colony spreading and extracellular proteolytic activity

**Figure 2:**
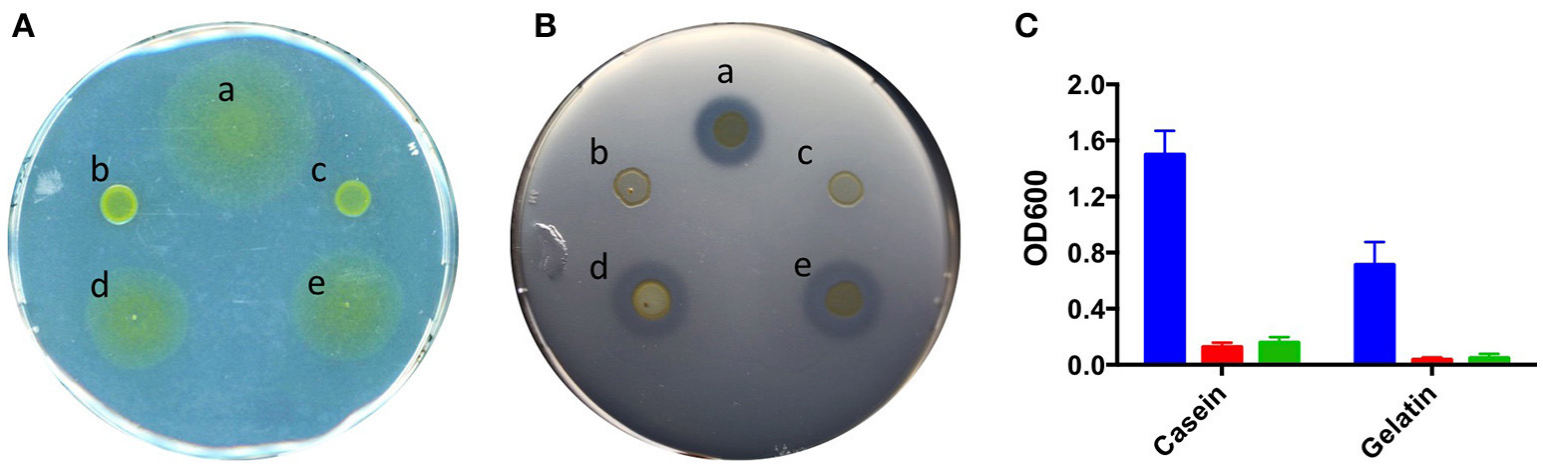
Spreading and extracellular proteolytic activity. **(A)** Spreading of five different strains of *F. psychrophilum* grown on 1/5 TYES agar after 72 h at 18°C: a, THCO2-90; b, *gldG::Tn*; c, *gldD::Tn;* d*, gldG::Tn* pCP*Gm*^*R*^-*gldG*; e*, gldD::Tn* pCP*Gm*^*R*^-*gldD*. **(B)** Extracellular proteolytic activity of the same strains grown on TYES agar + 0.75% gelatin after 72 h at 18°C. **(C)** Growth of strains THCO2-90 (blue), *gldG::Tn* (red), and *gldD::Tn (*green) in BH minimal broth medium supplemented with 0.5% of casein or gelatin after 72 h at 18°C, 200 rpm. The results are representative of three independent experiments.

The two mutant strains showed a lack of colony spreading when grown on 1/5 TYES agar, while the wild-type strain displayed the characteristic wide spreading phenotype (Figure 2A). In addition, strains *gldD::Tn* and *gldG::Tn* displayed a significant diminution of their extracellular proteolytic activity compared to the wild-type strain using gelatin as substrate (Figure 2B). Similar defects were observed using casein as substrate (data not shown). Interestingly, whereas no differences were observed in their growth kinetics when grown in the tryptone-rich TYES broth, with a doubling time of about 3 h for strains THCO2-90, *gldD::Tn* and *gldG::Tn*, both mutant strains were unable to grow in BH minimal broth supplemented with 0.5% casein or gelatin as C-source, contrary to the wild-type strain (Figure 2C).

### Complementation of *gldD::Tn* and *gldG::Tn* mutant strains

In order to complement the Tn*4351* erythromycin-resistant mutants, a new shuttle vector derived from pCP23 (Agarwal et al., 1997) was constructed by substituting the tetracycline-resistance marker *tetQ* by the *aac(6')-aph(2')* gentamycin-resistance gene. Conjugative transfer of the resulting plasmid, pCP*Gm*^*R*^, in *F. psychrophilum* led to hundreds of gentamycin-resistant clones after 3–4 days on TYES gentamycin. The presence of plasmid was confirmed by DNA extraction. No gentamycin-resistant clones were obtained using *E. coli* MFD*pir* empty of plasmid as donor. In contrast, conjugations performed using *E. coli* MFD*pir* pCP23 (*tetQ*) or empty of plasmid as donor strains led to hundreds of *F. psychrophilum* false positive clones, which appeared on TYES tetracycline after 6–7 days of incubation. To complement Tn*4351* mutants, the coding sequence of *gldD* and *gldG* was cloned under the control of a *F. psychrophilum* promoter into pCP*Gm*^*R*^ (see Figure S2). Introduction of pCP*Gm*^*R*^-*gldD* and pCP*Gm*^*R*^-*gldG* into *gldD::Tn* and *gldG::Tn* mutants, respectively, resulted in the complementation of each of them. Spreading on 1/5 TYES agar was comparable for the resulting colonies with those of the wild-type strain (Figure 2A). The extracellular proteolytic activity was also restored by plasmid introduction (Figure 2B). These results indicate that GldD and GldG are both required for efficient spreading and extracellular proteolytic activity in *F. psychrophilum*.

### Inactivation of *gldD* or *gldG* impairs adhesion, biofilm formation, and bacterial sedimentation

**Figure 3:**
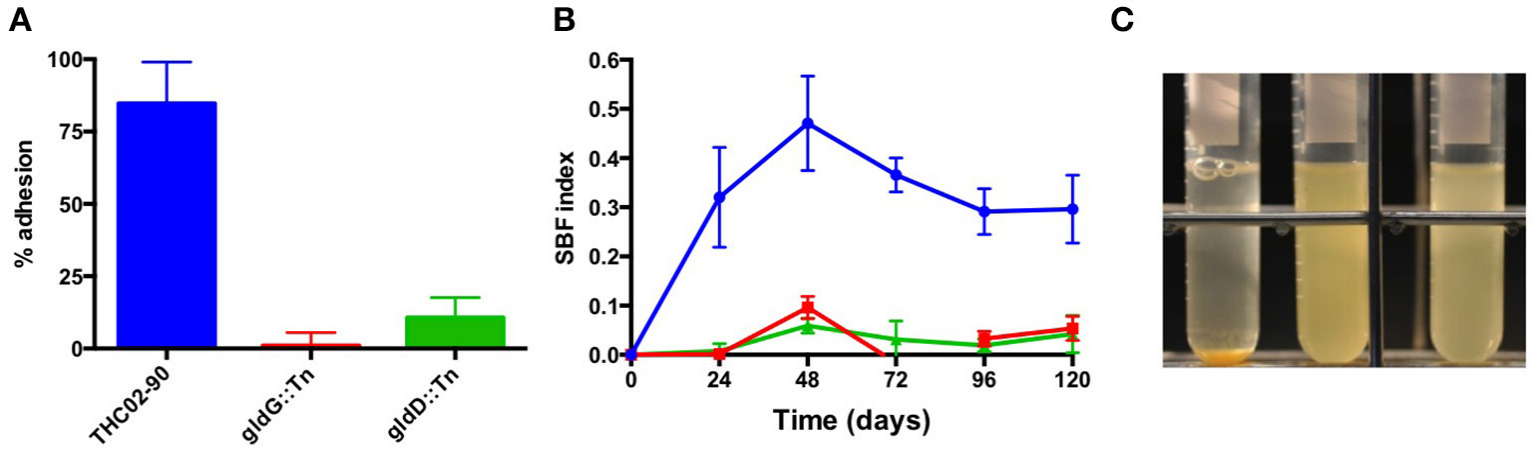
Adhesion, biofilm formation, and sedimentation. **(A)** Adhesion of the *F. psychrophilum* strains THCO2-90 (blue), *gldG::Tn* (red), and *gldD::Tn* (green) to polystyrene after 3 h of incubation at 18°C without shaking. **(B)** Biofilm formation kinetic of the same strains (same color code) grown in 1/2 TYES broth for 120 h at 18°C without shaking. **(C)** Bacterial cells sedimentation of strains THCO2-90 (left); *gldG::Tn* (center) and *gldD::Tn* (right) grown in TYES broth after 48 h at 18°C, 200 rpm. The results are representative of three independent experiments.

The ability of *F. psychrophilum* cells to adhere to polystyrene plates has been previously reported (Högfors-Rönnholm et al., 2015). Here, under similar assayed conditions, both strains *gldD::Tn* and *gldG::Tn* showed an impaired adhesion ability to polystyrene plates after 3 h of incubation at 18°C (Figure 3A). Biofilm formation depends on adhesion of microorganisms to each other and to biotic or abiotic surfaces and *F. psychrophilum* cells form biofilms on polystyrene plates (Álvarez et al., 2006; Sundell and Wiklund, 2011). Interestingly, both strains *gldD::Tn* and *gldG::Tn* showed a strongly reduced biofilm formation ability in contrast to the wild-type strain when grown in 1/2 TYES broth under static condition (Figure 3B). When grown as planktonic bacteria in liquid culture, reduced sedimentation was observed after 48 h of incubation for both mutant strains compared to the wild-type strain (Figure 3C). Altogether, these results imply that both GldD and GldG proteins are required for proper adhesion of *F. psychrophilum* cells to surfaces, as well as for auto-adhesion ability required for biofilm development.

### Inactivation of *gldD* or *gldG* impairs extracellular protein abundance

As described above, inactivation of *F. psychrophilum gldD* or *gldG* genes led to pleiotropic phenotypes *in vitro*. The impaired bacterial surface functions such as extracellular proteolytic activity, cell sedimentation and cell adhesion of strains *gldD::Tn* and *gldG::Tn* suggest an effect of these mutations on protein secretion efficiency. To test this hypothesis, we used a label-free proteomic approach that combined SDS-PAGE electrophoresis and LC-MS/MS analyses, to compare the exoproteomes of strains THCO2-90, *gldD::Tn* and *gldG::Tn* (Table S1). Among the 414 proteins identified in total, 90 and 158 were significantly altered in abundance in the culture supernatant of strains *gldD::Tn* and *gldG::Tn*, respectively, compared to those of wild-type strain. Among them, 20 and 9 proteins were not detected at all for strains *gldD::Tn* (Table 2) and *gldG::Tn* (Table 3), respectively, including the extracellular protease Fpp1 and a probable S8 subtilisin family serine endopeptidase. Strikingly, among the proteins significantly less abundant in the mutant strains, proteins involved in T9SS, gliding and other proteolytic enzymes were identified (i.e., GldN, GldK, and PorT that belong to the core T9SS machinery, the PorU peptidase; the SprB adhesin; the Fpp2 extracellular protease and the collagenase; Tables 2, 3). Among the 39 and 9 proteins predicted to have either a TIGR04183 or a TIGR04131 CTD in the proteome of strain THCO2-90, 29, and 5, respectively, were not found or significantly less abundant in both mutants' supernatants. Finally, 11 and 83 proteins were more abundant in the culture supernatant of strains *gldD::Tn* and *gldG::Tn*, respectively, compared to the wild-type strain. Among them, most are of unknown function, and none possess a CTD domain (Table S1).

**Table 2:** Secretome of strains THCO2-90 and *gldD::Tn* identified by LC-MS/MS analysis of cell-free supernatant^a^.

**Locus tag**	**Gene**	**Predicted function^b^**	**CTD^c^**	**Ratio TH/*gldD*^d^**	**Spectrum count averages^e^**	
					**TH**	***gldD*::Tn**
THC0290_0237	*fpp1*	Psychrophilic metalloprotease Fpp1 precursor	TIGR04183	Absent	17.33	0.00
THC0290_1494		Probable ribonuclease	TIGR04183	Absent	13.67	0.00
THC0290_1520		Probable endonuclease precursor	TIGR04183	Absent	10.67	0.00
THC0290_1527		Protein of unknown function precursor containing a C-terminal secretion signal. Putative adhesin	TIGR04131	Absent	10.33	0.00
THC0290_2201		Protein of unknown function precursor, putative adhesin		Absent	10.33	0.00
THC0290_1797		Protein of unknown function precursor	TIGR04183	Absent	9.00	0.00
THC0290_1998		Protein of unknown function precursor		Absent	7.67	0.00
THC0290_0944		Probable S8 subtilisin family serine endopeptidase precursor	TIGR04183	Absent	7.00	0.00
THC0290_1048		Protein of unknown function precursor, putative adhesin	TIGR04131	Absent	5.67	0.00
THC0290_0737		Protein of unknown function		Absent	4.00	0.00
THC0290_0121		Probable lipoprotein precursor		Absent	3.67	0.00
THC0290_0908		Protein of unknown function		Absent	3.67	0.00
THC0290_1054		Protein of unknown function precursor containing a C-terminal secretion signal	TIGR04183	Absent	3.00	0.00
THC0290_0171		Probable cell surface protein (Leucine-rich repeat protein) precursor	TIGR04183	Absent	8.33	0.00
THC0290_2158		Uncharacterized protein precursor. Probable phage protein.		54.00	18.00	0.33
THC0290_0500		Probable S8 and S53 subtilisin family serine endopeptidase precursor		52.67	52.67	1.00
THC0290_0743	*gldN*	Gliding motility protein precursor GldN		46.50	62.00	1.33
THC0290_0740	*gldK*	Gliding motility lipoprotein precursor GldK		43.50	58.00	1.33
THC0290_0339	*porT*	PorT protein		28.00	9.33	0.33
THC0290_0174		Probable cell surface protein (Leucine-rich repeat protein) precursor	TIGR04183	24.00	8.00	0.33
THC0290_2147	*gldJ*	Gliding motility lipoprotein precursor GldJ		23.67	94.67	4.00
THC0290_0754		Protein of unknown function precursor, putative adhesin		18.25	24.33	1.33
THC0290_1615		Protein of unknown function		18.00	24.00	1.33
THC0290_0291		Protein of unknown function precursor		18.00	6.00	0.33
THC0290_2029		Protein of unknown function precursor		17.50	11.67	0.67
THC0290_0129		Putative outer membrane protein precursor	TIGR04183	17.00	22.67	1.33
THC0290_0091		Probable glycoside hydrolase precursor	TIGR04183	14.00	9.33	0.67
THC0290_2146	*porU*	Por secretion system protein PorU precursor. C-terminal signal peptidase		12.25	16.33	1.33
THC0290_0931		Collagenase precursor	TIGR04183	11.29	26.33	2.33
THC0290_1526		Protein of unknown function precursor		10.00	3.33	0.33
THC0290_1595		Protein of unknown function precursor, putative adhesin	TIGR04183	9.75	13.00	1.33
THC0290_1616		Protein of unknown function precursor, putative adhesin	TIGR04131	8.33	16.67	2.00
THC0290_0175		Probable cell surface protein (Leucine-rich repeat protein)	TIGR04183	7.75	10.33	1.33
THC0290_0299		Probable M36 fungalysin family metalloprotease precursor	TIGR04183	6.93	106.33	15.33
THC0290_1047		Protein of unknown function precursor, putative adhesin	TIGR04131	6.50	4.33	0.67
THC0290_0176		Probable cell surface protein (Leucine-rich repeat protein) precursor	TIGR04183	6.40	10.67	1.67
THC0290_0238	*fpp2*	Psychrophilic metalloprotease Fpp2 precursor	TIGR04183	6.15	67.67	11.00
THC0290_0186		Probable cell surface protein (Leucine-rich repeat protein) precursor	TIGR04183	5.40	9.00	1.67
THC0290_0300		Probable M36 fungalysin family metalloprotease precursor	TIGR04183	5.00	6.67	1.33
THC0290_0173		Probable cell surface protein	TIGR04131	4.77	47.67	10.00

a *Proteins which relative abundance changed at least ±2-fold significantly (p < 0.01) and for which at least 3 spectra have been identified in one of analyzed strain (full data available in Table S1)*.

b *Proteins annotation as previously described (Rochat et al., 2017a)*.

c *CTD type identified by BLASTP analysis*.

d *Protein abundance ratio calculated using spectral counts*.

e *Average number of spectral counts calculated using triplicates values for each strain*.

**Table 3:** Secretome of strains THCO2-90 and *gldG::Tn* identified by LC-MS/MS analysis of cell-free supernatant^a^.

					**Spectrum count averages^e^**	
**Locus tag**	**Gene**	**Predicted function^b^**	**CTD^c^**	**Ratio TH/*gldG*^d^**	**TH**	***gldG*::Tn**
THC0290_0237	*fpp1*	Psychrophilic metalloprotease Fpp1 precursor	TIGR04183	Absent	17.33	0.00
THC0290_0944		Probable S8 subtilisin family serine endopeptidase precursor	TIGR04183	Absent	7.00	0.00
THC0290_1048		Protein of unknown function precursor, putative adhesin	TIGR04131	Absent	5.67	0.00
THC0290_0171		Probable cell surface protein (Leucine-rich repeat protein) precursor	TIGR04183	Absent	8.33	0.00
THC0290_2147	*gldJ*	Gliding motility lipoprotein precursor GldJ		40.57	94.67	2.33
THC0290_1520		Probable endonuclease precursor	TIGR04183	32.00	10.67	0.33
THC0290_0174		Probable cell surface protein (Leucine-rich repeat protein) precursor	TIGR04183	24.00	8.00	0.33
THC0290_0740	*gldK*	Gliding motility lipoprotein precursor GldK		17.40	58.00	3.33
THC0290_2201		Protein of unknown function precursor, putative adhesin		15.50	10.33	0.67
THC0290_0743	*gldN*	Gliding motility protein precursor GldN		13.29	62.00	4.67
THC0290_1047		Protein of unknown function precursor, putative adhesin	TIGR04131	13.00	4.33	0.33
THC0290_0500		Probable S8 and S53 subtilisin family serine endopeptidase precursor		10.53	52.67	5.00
THC0290_1615		Protein of unknown function		10.29	24.00	2.33
THC0290_1494		Probable ribonuclease	TIGR04183	10.25	13.67	1.33
THC0290_0129		Putative outer membrane protein precursor	TIGR04183	8.50	22.67	2.67
THC0290_0175		Probable cell surface protein (Leucine-rich repeat protein)	TIGR04183	7.75	10.33	1.33
THC0290_0238	*fpp2*	Psychrophilic metalloprotease Fpp2 precursor	TIGR04183	6.15	67.67	11.00
THC0290_2158		Uncharacterized protein precursor. Probable phage protein.		6.00	18.00	3.00
THC0290_0754		Protein of unknown function precursor, putative adhesin		5.62	24.33	4.33
THC0290_1616		Protein of unknown function precursor, putative adhesin	TIGR04131	5.56	16.67	3.00
THC0290_0300		Probable M36 fungalysin family metalloprotease precursor	TIGR04183	5.40	9.00	1.67

a *Proteins which relative abundance changed at least ±2-fold significantly (p < 0.01) and for which at least 3 spectra have been identified in one of analyzed strain (full data available in Table S1)*.

b *Proteins annotation as previously described (Rochat et al., 2017a)*.

c *CTD type identified by BLASTP analysis*.

d *Protein abundance ratio calculated using spectral counts*.

e *Average number of spectral counts calculated using triplicates values for each strain*.

Cell surface shaving with trypsin and LC-MS/MS analysis of strains *gldD::Tn* and wild-type enabled the identification of 426 proteins (Table S2). Among them, 74 were significantly altered in abundance in strain *gldD::Tn* compared to the wild-type strain. Indeed, 39 proteins (8 with a CTD domain) were not detected or significantly less abundant, including the GldJ gliding protein and the GldN T9SS machinery subunit (Table 4). In contrast, 35 proteins were more abundant in strain *gldD::Tn* such as the predicted cysteine protease FcpB and the gliding motility precursor RemF. Contrary to results obtained in the supernatant fractions, the SprB surface adhesin and the PorU peptidase were not detected in the wild-type cells, whereas these proteins were identified in strain *gldD::Tn* (Table 4).

**Table 4:** Surfome of strains THCO2-90 and *gldD::Tn* identified by LC-MS/MS analysis^a^.

**Locus tag**	**Gene**	**Predicted function^b^**	**CTD^c^**	**Ratio TH/*gldD*^d^**	**Spectrum count averages^e^**	
					**TH**	***gldD*::Tn**
THC0290_2147	*gldJ*	Gliding motility lipoprotein precursor GldJ		Absent	27.00	0.00
THC0290_2385		RCC1 (Regulator of Chromosome Condensation) repeat domain protein precursor	TIGR04183	Absent	5.67	0.00
THC0290_0143		ATPase, MoxR family		Absent	4.67	0.00
THC0290_0091		Probable glycoside hydrolase precursor	TIGR04183	Absent	4.33	0.00
THC0290_1535	*fahA*	Fumarylacetoacetase		Absent	4.33	0.00
THC0290_1494		Probable ribonuclease	TIGR04183	Absent	3.33	0.00
THC0290_1675		Probable asparagine synthetase [glutamine-hydrolyzing]		Absent	3.33	0.00
THC0290_0462	*nrdB*	Ribonucleoside-diphosphate reductase, beta subunit		Absent	3.00	0.00
THC0290_1326	*ybcL*	Probable phospholipid-binding protein precursor YbcL		Absent	3.00	0.00
THC0290_1520		Probable endonuclease precursor		Absent	3.00	0.00
THC0290_2305	*fabH3*	3-oxoacyl-[acyl-carrier-protein] synthase III protein FabH3		Absent	3.00	0.00
THC0290_0129		Putative outer membrane protein precursor	TIGR04183	45.00	15.00	0.33
THC0290_0149		Probable alcohol dehydrogenase		15.00	5.00	0.33
THC0290_0164	*sodA*	Superoxide dismutase [Mn]		15.00	5.00	0.33
THC0290_1498	*rho*	Transcription termination factor Rho		14.00	4.67	0.33
THC0290_1932		Protein of unknown function precursor containing a C-terminal secretion signal. Putative adhesin.	TIGR04131	12.67	12.67	1.00
THC0290_1081	*dapD*	2,3,4,5-tetrahydropyridine-2,6-dicarboxylate N-succinyltransferas		7.00	7.00	1.00
THC0290_0973	*lpdA1*	Dihydrolipoyl dehydrogenase		7.00	4.67	0.67
THC0290_0743	*gldN*	Gliding motility protein precursor GldN		6.80	11.33	1.67
THC0290_0754		Protein of unknown function precursor, putative adhesin		6.33	6.33	1.00
THC0290_2067	*rplQ*	50S ribosomal protein L17		5.33	5.33	1.00
THC0290_2146	*porU*	Por secretion system protein PorU precursor. C-terminal signal peptidase		0	0.00	22.67
THC0290_1795		Probable lipoprotein precursor		0	0.00	13.00
THC0290_0025	*sprB*	Putative adhesin precursor SprB (modular protein)	TIGR04131	0	0.00	6.33
THC0290_0021	*remF*	Gliding motility protein RemF precursor		0.30	3.67	12.33

a *Proteins which relative abundance changed at least ±2-fold significantly (p < 0.01) and for which at least 3 spectra have been identified in one of analyzed strain (full data available in Table S2)*.

b *Proteins annotation as previously described (Rochat et al., 2017a)*.

c *CTD type identified by BLASTP analysis*.

d *Protein abundance ratio calculated using spectral counts*.

e *Average number of spectral counts calculated using triplicates values for each strain*.

When analyzing the surfome of *gldG::Tn* compared to the wild-type strain, 582 proteins were identified in total (Table S2). Among the 69 proteins altered in abundance in strain *gldG::Tn*, 14 were not detected or significantly less abundant, including the above-mentioned GldJ, GldK, and GldN or the collagenase (Table 5), which is in agreement with the results obtained in the supernatant fractions (Table 3). In contrast, and as observed for strain *gldD::Tn*, 55 proteins were undetected or less abundant in the wild-type surfome, including SprB, PorU, and RemF (Table 5).

**Table 5:** Surfome of strains THCO2-90 and *gldG::Tn* identified by LC-MS/MS analysis^a^.

**Locus tag**	**Gene**	**Predicted function^b^**	**CTD^c^**	**Ratio TH/*gldG*^d^**	**Spectrum count averages^e^**	
					**TH**	***gldG*::Tn**
THC0290_2147	*gldJ*	Gliding motility lipoprotein precursor GldJ		Absent	27.00	0
THC0290_2385		RCC1 (Regulator of Chromosome Condensation) repeat domain protein precursor	TIGR04183	Absent	5.67	0
THC0290_0091		Probable glycoside hydrolase precursor	TIGR04183	Absent	4.33	0
THC0290_0232	*hppD*	4-hydroxyphenylpyruvate dioxygenase		Absent	3.67	0
THC0290_1494		Probable ribonuclease	TIGR04183	Absent	3.33	0
THC0290_1252		Universal stress protein, UspA family		Absent	3.00	0
THC0290_1520		Probable endonuclease precursor	TIGR04183	Absent	3.00	0
THC0290_2305	*fabH3*	3-oxoacyl-[acyl-carrier-protein] synthase III protein FabH3		Absent	3.00	0
THC0290_0740	*gldK*	Gliding motility lipoprotein precursor GldK		16.0	10.7	0.7
THC0290_0129		Putative outer membrane protein precursor	TIGR04183	11.3	15.0	1.3
THC0290_0743	*gldN*	Gliding motility protein precursor GldN		6.8	11.3	1.7
THC0290_0931		Collagenase precursor		2.7	33.7	12.3
THC0290_2146	*porU*	Por secretion system protein PorU precursor. C-terminal signal peptidase		0	0.00	18.33
THC0290_1795		Probable lipoprotein precursor		0	0.00	17.67
THC0290_0025	*sprB*	Putative adhesin precursor SprB (modular protein)	TIGR04131	0	0.00	8.33
THC0290_0946	*pafA*	Alkaline phosphatase precursor		0	0.00	8.00
THC0290_0866		Protein of unknown function		0	0.00	5.67

a *Proteins which relative abundance changed at least ±2-fold significantly (p < 0.01) and for which at least 3 spectra have been identified in one of analyzed strain (full data available in Table S2)*.

b *Proteins annotation as previously described (Rochat et al., 2017a)*.

c *CTD type identified by BLASTP analysis*.

d *Protein abundance ratio calculated using spectral counts*.

e *Average number of spectral counts calculated using triplicates values for each strain*.

This proteomic analysis reveals that inactivation of *gldD* or *gldG* by Tn*4351* results in a major defect in surface protein localization, especially for those proteins likely translocated through the T9SS apparatus.

### Virulence of *F. psychrophilum* is impaired by mutation in *gldD* or *gldG* in a rainbow trout infection model

**Figure 4:**
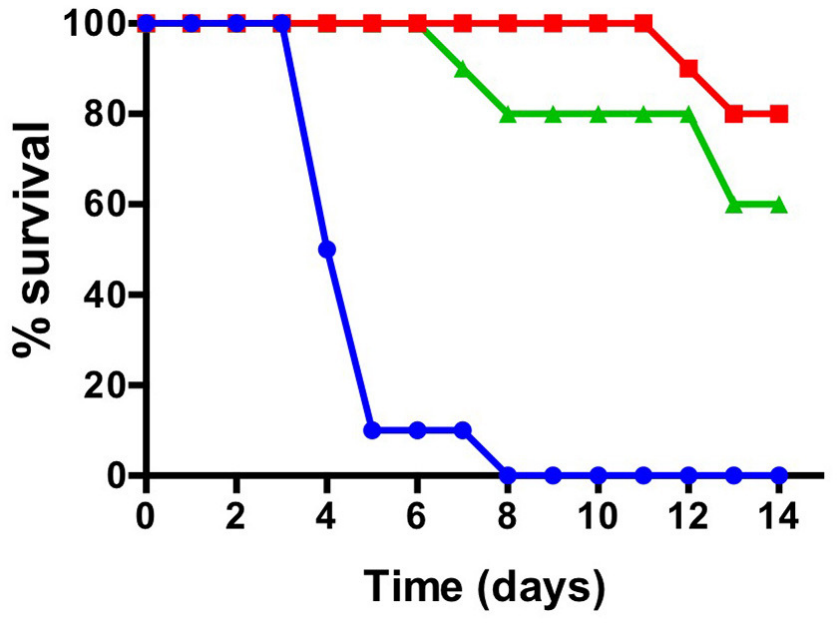
Intramuscular experimental challenge. Rainbow trout survival following intramuscular injection with *F. psychrophilum* strains THCO2-90 (blue), *gldG::Tn* (red), and *gldD::Tn* (green). The plots show the survival of rainbow trout following intramuscular challenge with 2 × 10^4^ CFU of each strain for 14 days. The results are representative of two independent experiments.

The effect of *gldD* or *gldG* inactivation on the virulence of *F. psychrophilum* was investigated in rainbow trout using two different routes of infection, intramuscular injection and immersion. First, the LD_50_ of strain THCO2-90 was established at 6.0 × 10^3^ bacteria by performing intramuscular injection challenge in rainbow trout. This value was used as a reference to compare the virulence of strains THCO2-90, *gldD::Tn* and *gldG::Tn*. Groups of 10 fish were injected with 2 x 10^4^ CFU of strains THCO2-90, *gldD::Tn* or *gldG::Tn*. This dose theoretically corresponds to 4-fold the LD_50_ of the wild-type strain. Fish challenged with the wild-type strain quickly died and the cumulative mortality reached 100% 8 days post infection. In contrast, strains *gldD::Tn* and *gldG::Tn* showed cumulative mortalities of 20 and 0% respectively by 8 days, and 40 and 20% by 14 days (Figure 4).

**Figure 5:**
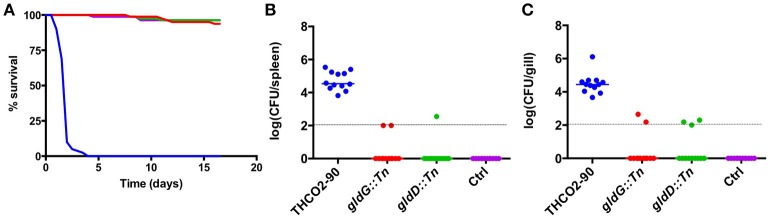
Experimental immersion challenge. **(A)** Rainbow trout survival following infection by immersion challenge with *F. psychrophilum*. Fish were infected for 24 h with 5 × 10^6^ CFU mL^−1^ in a final volume of 15 L with strains THCO2-90 (blue), *gldG::Tn* (red), and *gldD::Tn* (green). **(B,C)** Bacterial load of rainbow trout infected with strains THCO2-90, *gldG::Tn* and *gldD::Tn*. Six fish infected by each strain were sacrificed at 4 h post-infection. The bacterial loads of spleen **(B)** and gills **(C)** are shown. The results of two independent experiments are presented.

We further investigated the impact of *gldD* or *gldG* inactivation on virulence using an immersion challenge model that supposedly more closely mimics the natural infection route. Fish were bathed for 24 h in water contaminated by bacterial culture of strains THCO2-90, *gldD::Tn* or *gldG::Tn* (theoretical initial concentration 5 × 10^6^ CFU mL^−1^). Following the bath challenge, an increase in bacterial concentration in water was observed for all tanks, whatever the strain (Table 6). Four days post-infection, fish cumulative mortality reached 100% with the wild-type strain, while the mortality of fish infected with strains *gldD::Tn* or *gldG::Tn* was similar to that of the non-infected group (≤8%; Figure 5A). When bath-challenged with the wild-type strain, the bacterium was detected on the surface and in organs of all fish arbitrarily sampled 6-h after the end of immersion. The bacterial load in spleen and gills was on average 3.5 × 10^4^ and 2.8 × 10^4^ CFU, respectively. In contrast, the bacterium was only detected in 2 out of 12 fish infected with strains *gldD::Tn* or *gldG::Tn*. For those fish, the bacterial loads were 10^2^ and 3.5 × 10^2^ CFU in the spleen and 1.4 × 10^2^ and 2.6 × 10^2^ CFU in the gills, respectively (Figures 5B,C). In addition, *F. psychrophilum* was always detected in samples of skin mucus of fish infected with the wild-type strain, while the bacterium was systematically absent from the skin mucus of fish infected with the mutant strains (12 fish sampled for each group; data not shown).

**Table 6:** Bacterial loads in aquarium water during immersion challenge.

**CFU mL**^**−1**^		
	**Time (hours)**	
**Strain**	**0**	**24**
THCO2-90	2 × 10^6^	8 × 10^7^
*gldD::Tn*	9 × 10^6^	4 × 10^7^
*gldG::Tn*	8 × 10^6^	5 × 10^7^

*Average of bacterial quantification determined at the beginning (0 h) and the end (24 h) of fish infection challenge from two independent experiments*.

These results revealed that the virulence of strains *gldD::Tn* and *gldG::Tn* is strongly attenuated in rainbow trout whatever the infection route, with considerably lower invasion and proliferation abilities *in vivo*.

## Discussion

In order to understand the role and the relative importance of *F. psychrophilum* genes in its lifestyle, a Tn*4351-*mutant library was constructed in the strain THCO2-90. By characterizing a subset of this library by inverse-PCR, two *F. johnsoniae* orthologous genes, *gldD* and *gldG*, were found disrupted in our mutant collection. Both *gldD::Tn* and *gldG::Tn* display a lack of spreading on agar plate which is restored by complementation. In addition to their motility deficiency, other phenotypes such as impaired extracellular proteolytic activity or reduced adhesion ability *in vitro* and *in vivo* were observed. In *F. johnsoniae*, strains carrying mutations in *gldD* or *gldG* also exhibit phenotypes other than motility such as extracellular chitin utilization deficiency and a higher resistance to bacteriophage infections (Hunnicutt and McBride, 2001; Hunnicutt et al., 2002). In addition, a *gldD* mutant in *C. algicola* showed only partially deficient motility (i.e., residual spreading on agar still occurred), which raised the idea that GldD may have a role other than gliding (Zhu and McBride, 2016).

The deficiency in extracellular proteolytic activity of strains *gldD::Tn* and *gldG::Tn* in *F. psychrophilum* suggests that inactivation of these genes has consequences on the secretion process. Bacterial exoproteome analyses showed important changes in the abundance of extracellular proteins in both mutants. The disruption of the *gldD* or *gldG* gene of *F. psychrophilum* provokes a remarkable diminution of GldJ gliding protein. Similarly, in *F. johnsoniae*, disruption of the orthologous genes *gldD* and *gldG* resulted in a dramatic reduction of GldJ abundance (Braun and McBride, 2005). In addition, the disruption of *gldD* or *gldG* provokes a remarkable diminution of GldK, GldN, and SprT proteins, described as three components of the core machinery of the T9SS in *F. johnsoniae* (Rhodes et al., 2010; Shrivastava et al., 2013), *C. ochracea* (Kita et al., 2016) and *P. gingivalis* (Sato et al., 2010, 2013). These changes might explain the secretion deficiency observed in *gldD* or *gldG* mutants. Strikingly, among the proteins absent or significantly less abundant in the exoproteome of both mutant strains, a significant number possess one of the TIGR04183 or TIGR04131-type domains, which were found to be required for efficient protein export by the T9SS in several *Bacteroidetes* species (McBride and Nakane, 2015; Kulkarni et al., 2017). It is important to note that not all proteins follow this trend. The SprB adhesin, which carries a TIGR04131-type domain required for its secretion to the cell surface by the T9SS (Rhodes et al., 2010; Sato et al., 2010; Shrivastava et al., 2013) is less abundant in the spent media of mutant strains compared to the wild-type strain. However, opposite results were observed in the surfome analyses: SprB was not detected on wild-type cells surface, whereas specific peptides were detected in mutant strains. Surfome proteomic analysis was performed using the intact cell surface shaving method, which is based on the digestion of exposed proteins by added trypsin, resulting in peptides release for further identification. One key limitation to this approach is that only the protruding protein domains are accessible to proteases (Grandi, 2010). The differences observed in the case of SprB could be attributed to technical limitations, such as a protein resistance to trypsin cleavage under these native conditions or a hindered access to trypsin. In *F. johnsoniae*, the deletion of PorV showed that the secretion of many but not all proteins carrying the T9SS C-terminal secretion signal was affected. It should to be noted that the secretion of SprB was not disturbed (Kharade and McBride, 2015). Recently, it has been proposed that the secretion of SprB-like large proteins (366 kDa) may require the involvement of additional proteins in addition to those forming the T9SS, as well as regions of the secreted protein other than the CTD to interact with the secretion system (Kulkarni et al., 2017). Altogether, our results suggest that mutations in *gldD* or *gldG* provoke a perturbation in the T9SS activity of *F. psychrophilum*. The exact reasons for the disorders observed in mutant strains remain unknown and further studies are needed to determine the precise molecular functions of GldD and GldG proteins. They could be involved in any critical steps of the secretion process, from the T9SS assembly to the recognition and translocation of its substrates across the outer membrane, or they may be involved in still another unknown function that indirectly impacts the T9SS. The decreased amount of three components of the core machinery in both mutants could result in a bottleneck, limiting protein export and possibly explaining the reduced secretion efficiency observed using proteomics. Taking into account these results, it is also unclear whether strains *gldD::Tn* and *gldG::Tn* are defective in spreading because they lack the gliding motor or due to an inefficient protein secretion.

Undoubtedly, the pleiotropic effects displayed by the *gldD::Tn* and *gldG::Tn* mutants have a profound impact on their virulence as shown by their very low ability to colonize fish and to induce mortality using rainbow trout as an infection model. The immersion challenge revealed the importance of GldD and GldG in *F. psychrophilum* virulence. In contrast with fish infected with the wild-type strain, both mutant strains were not detected in the skin mucus of experimentally infected fish and they showed a lower ability to colonize the gills. These results are in line with the important diminution of adhesive properties observed *in vitro*. Skin mucus acts as a first barrier and is an important part of the fish immune system, containing numerous antibacterial factors secreted by skin cells, such as immunoglobulins, agglutinins, lectins, lysins, and lysozymes; but skin mucus can also be an important microenvironment and portal of entry for pathogenic bacteria (Benhamed et al., 2014). *F. psychrophilum* is able to adhere to fish skin mucus (Högfors-Rönnholm et al., 2015) and to different mucosal tissues such as fins, gills, skin, and eyes (Nematollahi et al., 2003b; Papadopoulou et al., 2017). Exoproteome analyses revealed that both mutant strains display an important decrease in the amount of several putative adhesins. Papadopoulou and co-workers had previously noted that the *F. psychrophilum* adhesion process was likely mediated by bacterial surface proteinaceous compounds, since proteinase K treatment of cells significantly decreased bacterial adhesion to polystyrene surface (Papadopoulou et al., 2015). The lack of one or some of these adhesins is thus likely responsible for the impaired adhesion of mutant cells to abiotic or biotic surfaces. Both mutant strains exhibit also an impaired biofilm formation capacity. It is well-known that adhesion to surfaces is the first essential step during microbial biofilm formation (Joo and Otto, 2012). Our results are consistent with findings describing a significant overexpression of genes encoding some of these predicted adhesins (e.g., THC0290_1047, THC0290_1048, and THC0290_2201) during biofilm formation in other *F. psychrophilum* strains (Levipan and Avendaño-Herrera, 2017). Some of these adhesins possess a TIGR04183 or TIGR04131 domain. These observations indicate a potential requirement of T9SS for secretion of adhesion factors and, consequently, for biofilm formation by *F. psychrophilum*, as previously demonstrated in *F. johnsoniae* (Shrivastava et al., 2013) and in *C. ochracea* (Kita et al., 2016).

The lack of extracellular proteolytic activity found *in vitro* by both strains *gldD::Tn* and *gldG::Tn* could explain their high degree of attenuation during rainbow trout infection. Proteolytic degradation of host tissues has been reported to be involved in the virulence of several fish-pathogenic bacteria such as *Yersinia ruckeri, Vibrio anguilarum*, and *Edwarsiella tarda* (Fernandez et al., 2003; Yang et al., 2007; Zhou et al., 2015), and secreted enzymes were also proposed as virulence factors in *F. psychrophilum* (Bertolini et al., 1994; Ostland et al., 2000). One of the most reduced extracellular protease in the exoproteome of mutant strains, the collagenase, was found to be involved in the virulence of *F. psychrophilum* in ayu (*Plecoglossus altivelis*) as a fish infection model (Nakayama et al., 2015). Other extracellular proteases such as two M36 fungalysin family metalloproteases or the subtilisin-like extracellular protease were much less abundant in mutant strains and they also may contribute to virulence. Interestingly, it has been reported that the inactivation by Tn*4351* of *fpgA* encoding a glycosyltransferase results in similar phenotypes as *gldD* or *gldG* inactivation, such as a lack of colony spreading and extracellular proteolytic activity and a complete loss of virulence (Pérez-Pascual et al., 2015).

The role of GldD and GldG proteins was first studied in the environmental *Bacteroidetes* species *F. johnsoniae* and was long considered to be essentially linked to gliding motility (Hunnicutt and McBride, 2001; Hunnicutt et al., 2002). The previous observation that the non-gliding bacterium *P. gingivalis* lacks *gldD* and *gldG* orthologs also suggested that they might be involved more in gliding than in secretion. However, our results do not support this assumption. In this study, we assessed for the first time their role in the fish pathogen *F. psychrophilum*. The exhaustive exoproteome analysis of *gldD* and *gldG* mutants highlighted their impact on the secretory process, especially for some but not all proteins translocated by the T9SS. Extracellular proteins are of particular interest as they provide insight into the pathogenicity of this microorganism. Our data support the hypothesis that the impaired secretion of extracellular enzymes and adhesins resulting from the disruption of *gldD* or *gldG* is responsible for the reduced host colonization and infective ability of *F. psychrophilum*. These results provide important information when considering the lack of knowledge regarding *F. psychrophilum* virulence factors as well as the need to identify new targets for therapeutic interventions against *F. psychrophilum* infections.

## Author contributions

DP-P: Mutant library construction, phenotypic characterization, proteomics, animal experimentation, and drafting of the manuscript; TR: phenotypic characterization, mutant complementation, animal experimentation, and drafting of the manuscript with substantial intellectual contribution; BK: mutant library construction and animal experimentation; EG and FN-R: mutant library construction; CH: proteomics (sample preparation, LC-MS/MS, statistical analyses of data); EQ: development of the rainbow trout isogenic line and data interpretation; JG: data analysis and manuscript preparation; JB: substantial intellectual contribution throughout the study, animal experimentation, data analysis and manuscript preparation. ED: substantial intellectual contribution throughout the study, interpretation of data, manuscript preparation, and acquisition of funding. All authors read and approved the final manuscript.

### Conflict of interest statement

The authors declare that the research was conducted in the absence of any commercial or financial relationships that could be construed as a potential conflict of interest.
