# Supplementary figures and images for: More Than Gliding: Involvement of GldD and GldG in the Virulence of Flavobacterium psychrophilum
Source: Front Microbiol. 2017 Nov 7;8:2168. doi: 10.3389/fmicb.2017.02168 (PMC5682007; doi:10.3389/fmicb.2017.02168)

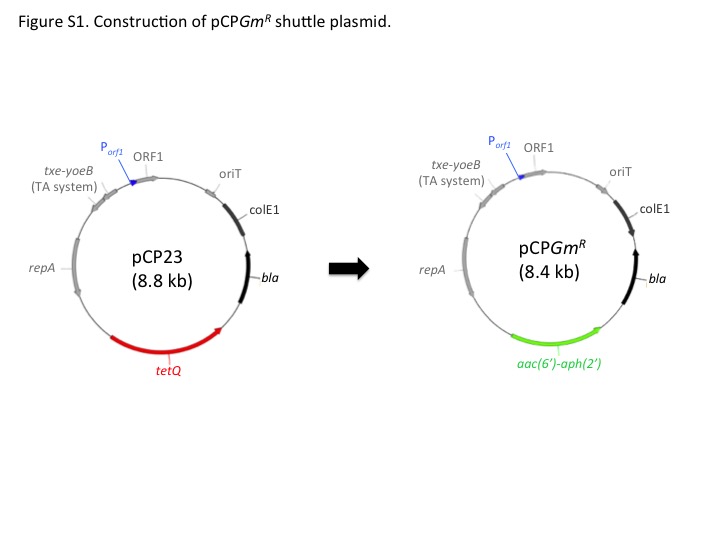

Supplement: Supplementary file 1 [file Image1.JPEG]

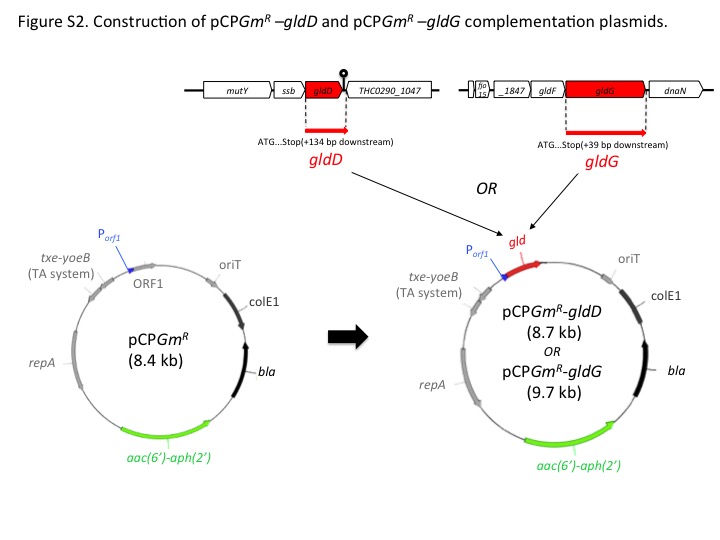

Supplement: Supplementary file 2 [file Image2.JPEG]

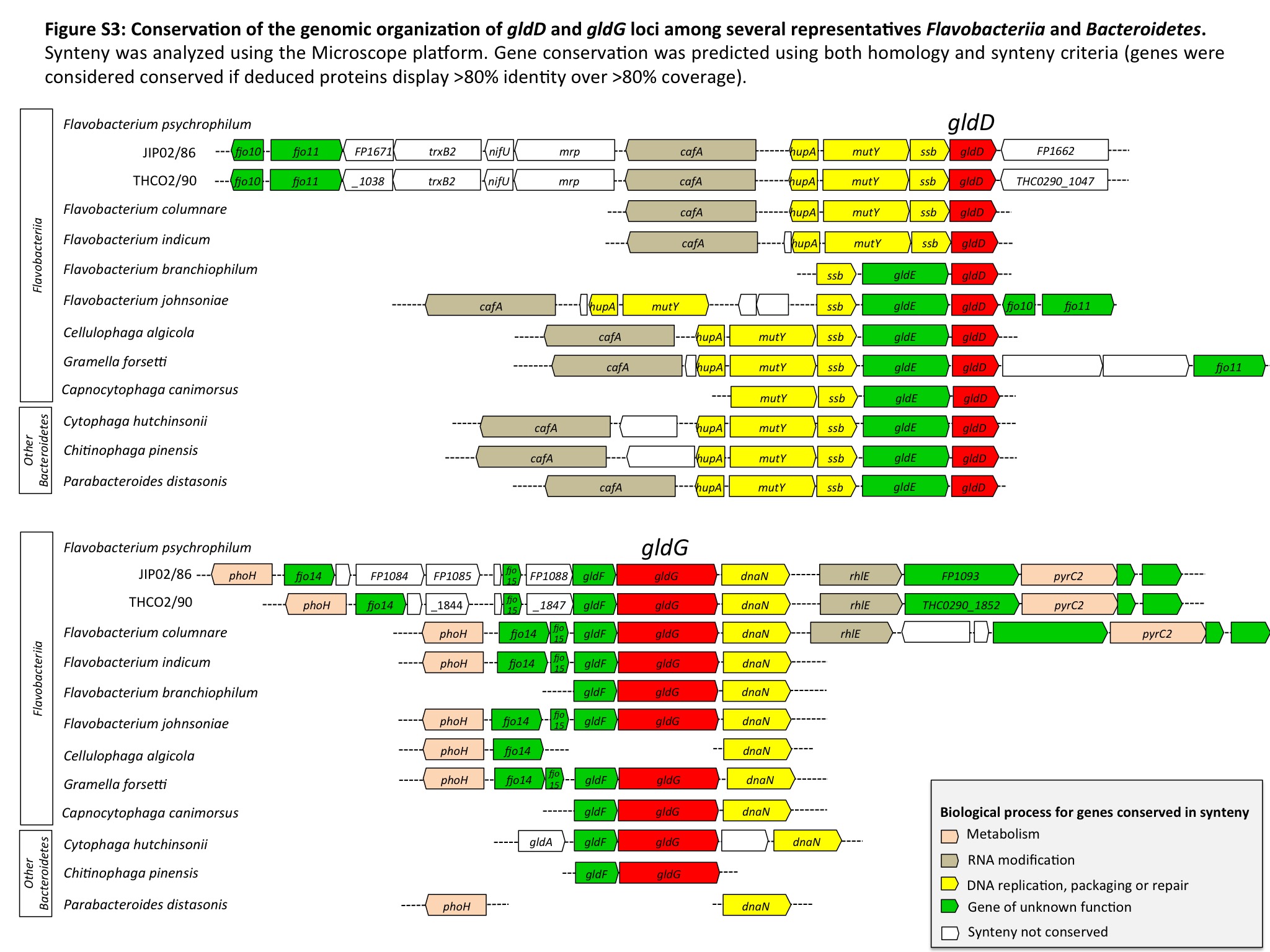

Supplement: Supplementary file 3 [file Image3.jpg]
